# Identifying the Characteristics of Responders and Nonresponders in a Behavioral Intervention to Increase Physical Activity Among Patients With Moderate to Severe Asthma: Protocol for a Prospective Pragmatic Study

**DOI:** 10.2196/49032

**Published:** 2023-08-31

**Authors:** Fabiano Francisco de Lima, Adriana Claudia Lunardi, David Halen Araújo Pinheiro, Regina Maria Carvalho-Pinto, Rafael Stelmach, Pedro Giavina‑Bianchi, Rosana Câmara Agondi, Celso RF Carvalho

**Affiliations:** 1 Department of Physical Therapy School of Medicine University of São Paulo São Paulo Brazil; 2 Pulmonary Division Instituto do Coração, School of Medicine University of São Paulo São Paulo Brazil; 3 Department of Clinical Immunology and Allergy School of Medicine University of São Paulo São Paulo Brazil

**Keywords:** asthma, behavioral intervention, physical activity, responders

## Abstract

**Background:**

Previous research has suggested that most adults improve their asthma control after a short-term behavioral intervention program to increase physical activity in daily life (PADL). However, the characteristics of individuals who respond and do not respond to this intervention and the medium-term response remain unknown.

**Objective:**

This study aims to (1) identify the characteristics of adult responders and nonresponders with asthma to a behavioral intervention to increase physical activity and (2) evaluate the functional and clinical benefits in the medium term.

**Methods:**

This prospective pragmatic study will include adults with moderate to severe asthma who enroll in a behavioral intervention. All individuals will receive an educational program and an 8-week intervention to increase PADL (1 time/wk; up to 90 min/session). The educational program will be conducted in a class setting through group discussions and video presentations. Behavioral interventions will be based on the transtheoretical model using counseling, incentives, and individual feedback aiming to increase participation in physical activity. Motivational interviewing and guidelines for overcoming barriers will be used to stimulate individuals to reach their goals. Pre- and postintervention assessments will include the following: PADL (triaxial accelerometry), body composition (octopolar bioimpedance), barriers to PADL (questionnaire), clinical asthma control (Asthma Control Questionnaire), quality of life (Asthma Quality of Life Questionnaire), anxiety and depression levels (Hospital Anxiety and Depression Scale), and exacerbations. “Responders” to the intervention will be defined as those who demonstrate an increase in the number of daily steps (≥2500).

**Results:**

In December 2021, the clinical trial registration was approved. Recruitment and data collection for the trial is ongoing, and the results of this study are likely to be published in late 2024.

**Conclusions:**

The intervention will likely promote different effects according to the clinical characteristics of the individuals, including asthma control, age, anxiety and depression levels, obesity, and several comorbidities. Identifying individuals who respond or do not respond to behavioral interventions to increase PADL will help clinicians prescribe specific interventions to adults with asthma.

**Trial Registration:**

ClinicalTrials.gov NCT05159076; https://clinicaltrials.gov/ct2/show/NCT05159076

**International Registered Report Identifier (IRRID):**

DERR1-10.2196/49032

## Introduction

According to the Global Initiative for Asthma (GINA), asthma is a heterogeneous disease characterized by chronic airway inflammation [1]. Asthma is defined by the history of respiratory symptoms, such as shortness of breath, wheezing, chest tightness, and cough, which can vary over time and in intensity, together with variable expiratory airflow limitation [1]. Asthma is a global health problem that affects all age groups. For example, in 2019, about 262 million people were diagnosed with asthma, and 461,000 died from this disease [2]. Moreover, its prevalence is increasing, affecting up to 18% of the population in some countries [1].

Several treatments, both pharmacological and nonpharmacological, are involved in the control of asthma. A supervised, structured exercise program is a nonpharmacological treatment known to improve asthma control [3]. However, despite the proven benefits of exercise, several barriers, such as the lack of space, limited access to pulmonary rehabilitation programs, and decreased motivation to remain physically active after the completion of such programs [4], make interventions to increase physical activity in daily life (PADL) an attractive and viable option [5].

Despite the known benefits of being physically active that have already been shown in this population, individuals with asthma are less physically active than control populations [6]. For instance, a previous study demonstrated that physical inactivity and a sedentary lifestyle are the only extrapulmonary factors associated with asthma exacerbation [7]. In addition, higher levels of physical activity positively impacted the clinical outcomes in this group [6]. Consequently, it is important to develop strategies to modify behavior to increase physical activity and reduce sedentary activities in this population.

The World Health Organization (WHO) recommends that adults perform 150 to 300 minutes of moderate-intensity aerobic physical activity weekly to achieve certain health benefits [8]. In addition, international guidelines [9] and GINA [1] classify, with A-level evidence, people with asthma who engage in regular physical activity to reduce cardiovascular risk and improve their quality of life [1]. A previous study found that a behavioral intervention to increase PADL improves clinical asthma control, sleep quality, and anxiety symptoms in adults with moderate to severe asthma [5]. However, 17% of the individuals did not improve their physical activity level, and 25% did not improve their clinical control after the intervention. Individuals with other chronic respiratory [10], cardiac [11], musculoskeletal [12], and neurological diseases [13] have also been classified as better or worse responders to specific interventions.

Thus, despite the benefits obtained by behavioral intervention aimed at increasing PADL in adults with asthma [5], it is necessary to know the characteristics of responders and nonresponders to guide the management in a more personalized approach. Moreover, the effects of this intervention were only assessed in the short term (immediately after the intervention), and the medium-term effects remained unknown. We hypothesize that the intervention might cause different effects according to the clinical characteristics of the individuals, including asthma control, age, anxiety and depression levels, obesity, and several comorbidities. Therefore, the main objective this prospective cohort study will be to identify the characteristics of adult responders and nonresponders with moderate to severe asthma to a behavioral intervention to increase PADL and evaluate the functional and clinical responses in the short and medium terms.

## Methods

### Study Design

**Figure 1:**
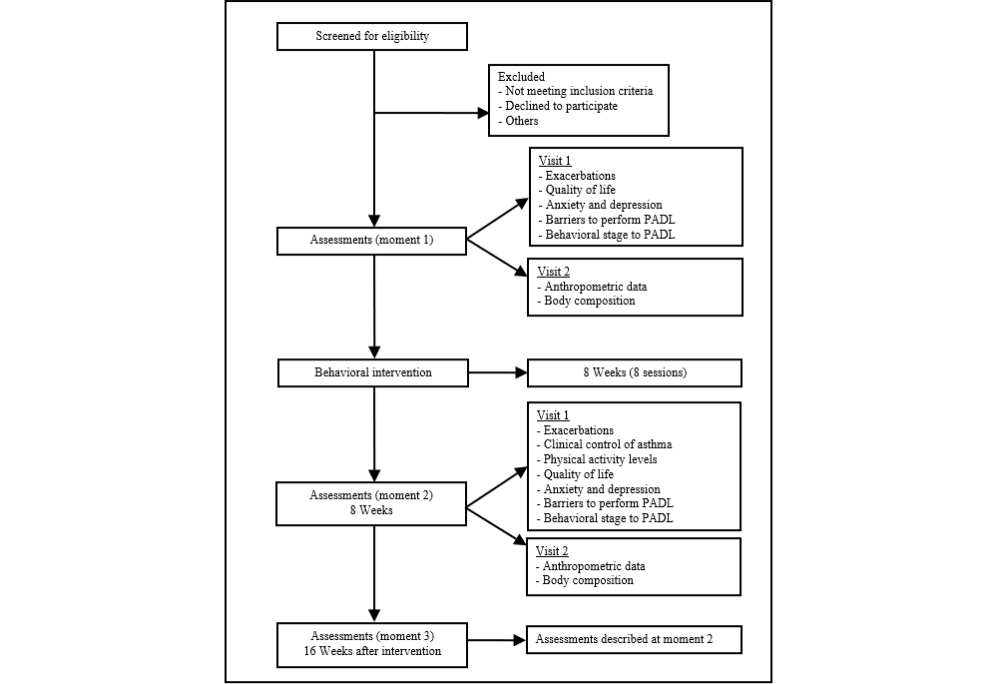
Study design. PADL: physical activity in daily life.

This prospective cohort study will be conducted at a university hospital that provides specialized asthma treatment. The study protocol was developed following the SPIRIT (Standard Protocol Items: Recommendations for Interventional Trials) checklist guidelines [14], and the trial was registered at ClinicalTrials.gov (NCT05159076). The study design is illustrated in Figure 1. Individuals with asthma will be enrolled in the trial after a routine medical consultation. Baseline lung function will be checked from the medical records. Eligible participants will undergo an 8-week behavioral intervention protocol to increase PADL and reduce sedentary behavior. First, all included individuals will receive an educational program, followed by an intervention aimed at increasing physical activity. All individuals will be assessed before and after the 8-week intervention and then 16 weeks later. The outcomes assessed will include anthropometric variables, clinical asthma control, health-related quality of life, physical activity levels, anxiety and depression symptoms, exacerbations, body composition, barriers to performing PADL, and physical activity behavioral stages (Figure 1).

### Participants

The study will include both male and female adults aged 18 to 65 years with moderate to severe asthma [1] who are not physically active (<150 min of moderate to vigorous physical activity/wk [15] or ≤7500 steps/d) and have partially controlled or uncontrolled asthma (Asthma Control Questionnaire [ACQ] score ≥0.75) [16]. In addition, the following inclusion criteria will be considered: diagnosis of asthma based on the recommendations established by GINA [1], outpatient medical treatment for at least six months, and a stable clinical status for at least 30 days (no hospitalizations, visits to emergency services, or medication changes) [17]. Pharmacotherapy will be maintained throughout the intervention period. After being informed about the possible risks and benefits of the study, participants will be instructed to sign an informed consent form.

### Ethics Approval

This study will be performed in accordance with the Declaration of Helsinki. Ethics approval has been granted by the Research Ethics Committee of the Hospital das Clínicas da Faculdade de Medicina da Universidade de São Paulo (#51929221.7.0000.0068). 

### Consent to Participate

Written informed consent will be obtained by researchers from all study participants.

### Analysis of the Population

Exclusion criteria will include participation in another research study, difficulty understanding any questionnaires, and pregnancy or psychiatric problems that make it difficult to understand the questionnaires and study protocol. If a patient misses any session, they will be contacted by telephone to confirm the reason, and the session will be rescheduled. Attending a total of 6 sessions will be considered the cut-off point to establish patient compliance [18-20]. An intention-to-treat analysis will be performed using the patient’s most recent assessment in the case of withdrawal from the study or the absence of data. The intervention will be conducted by a trained physiotherapist who is not otherwise directly involved in the assessments. In case of possible injuries occurring during the intervention period, the individuals will be referred for appropriate treatment.

### Procedures

#### Interventions

The intervention proposed here has been previously performed for individuals with asthma [5]. All individuals will participate in a brief asthma education program with 2 sessions, each lasting approximately 90 minutes. The educational program will be conducted in a class setting in the form of group discussions and video presentations. Educational topics will include asthma pathophysiology, medication and peak flow meter use, self-monitoring symptoms, environmental hygiene [1,21], and physical activity recommendations and benefits [15].

Behavioral interventions will be based on the transtheoretical model using counseling and incentives to increase the practice of physical activity [22,23]. Motivational interviewing, feedback, and guidelines for overcoming barriers will be used to stimulate individuals to reach their goals. Furthermore, individuals will receive a commercially available smartwatch activity monitor (Mi Band 5; Xiaomi) with an alarm that vibrates when the recommended daily number of steps is achieved and if the individual remains sedentary for 60 minutes. The main objective of the behavioral intervention will be to increase the level of PADL at any intensity, and the secondary objective will be to reduce sedentary time. The program will include 8 weekly face-to-face individual sessions, lasting up to 90 minutes each. At the beginning of the protocol, the motivational interview will identify the behavioral stage for physical activity practice using an appropriate questionnaire [24]. In addition, individuals will be asked to complete a daily physical activity diary and sign a contract with the health professional.

The data referring to the respective weeks of use will be reviewed for each behavioral intervention session. Each individual will receive advice based on their smartwatch data. Each session will address different topics related to physical activity practice and sedentary behavior, and individuals will be motivated and encouraged to make behavioral changes. In the last session, a motivational interview will be conducted to identify the individual’s behavioral stage, goals achieved, benefits gained, strategies used to overcome the most significant barriers, and proposed attitudes to keep themselves physically active in the long term. The schedule and content of each session are detailed in Table 1. The protocol of Freitas et al [25] will be modified by only changing the activity smartwatch monitor used during the intervention (Mi Band instead of Fitbit) [25].

**Table 1:** Description of the behavioral change intervention sessions. Reproduced from Freitas et al [25] under CC-BY License [26] . In this study, Mi Band 5 replaces Fitbit Flex 2.

No. Week	Topics covered
Week 1: Lifestyle choices	Motivational interview to establish an educational diagnosis Identify the behaviour stage regarding physical activity Raise awareness of physical activity benefits Provide the Fitbit Flex 2 and ask them to wear it for at least 3 days each week
Week 2: Why become physically active?	Raise awareness of the physical activity international recommendations Deepen the knowledge about the physical activity benefits for patients with asthma Review Fitbit Flex 2 data of the past week and set one smart weekly goal (number of steps) Establish the action planning (goal) and sign a contract Evaluate the confidence of patients in achieving the goal (self-efficacy) Explain about the use of the workbook, diary and vibration alert
Week 3: Sedentary behaviour	Raise awareness of the risks of prolonged uninterrupted periods of sitting Ask them to start monitoring their sitting time (diary in a workbook) Discuss strategies to stand up/break up the sedentary time, according to the Fitbit Flex 2 vibration function Review achievement (using the diary and Fitbit Flex 2 data) of the current goal Discuss progress of the current goal Set one smart weekly goal (number of steps)
Week 4: Dealing with barriers	Dealing with barriers (as part of action and coping planning) Brainstorm the main barriers and possible solutions/modifications Discuss preferred activities Invite participants to come up with ideas for walking (progression in duration/intensity) Congratulate patients on any success (positive reinforcement) and ask them to reflect on any difficulties Review achievement (using the diary and Fitbit Flex 2 data) of the current goal Progress the current smart goal (number of steps and sedentary behaviour)
Week 5: Self-control	Facilitate self-control (how to self-monitor the negative and positive behaviours regarding PA) Identify the benefits acquired with the lifestyle change and reinforce the commitment to change Invite participants to come up with ideas to break up the sedentary time Review achievement (using the diary and Fitbit Flex 2 data) of the current goal Progress current goal(s) as able/required
Week 6: Setting additional goal	Review initial goal and discuss the progress of this goal (challenges) Evaluate the confidence about achieving the new goal (self-efficacy) Reinforce the health benefits of increased participation in PA and of breaking up sedentary time Congratulate any success and reflect on any difficulties Review achievement (using the diary and Fitbit flex 2 data) of the current goal Set a new smart goal as able/required
Week 7: Being rewarded	Identify the behaviour stage regarding physical activity Discuss the change (or not) that was achieved, as well as the benefits acquired with the new lifestyle Discuss positive reinforcement Review achievement (using the diary and Fitbit flex 2 data) of the current goal Set a final goal (number to steps)
Week 8: Goal balance	Final motivational interview (goal setting, benefits acquired and strategies to overcome barriers) Reinforce the importance of following through with these changes Establish a long-term goal to stay physically active

#### Participant Timeline

The schedule of enrollment, interventions, and assessments is outlined in Table 2. The recruitment of the study participants began in May 2022.

**Table 2:** Content for the schedule of enrollment, interventions, and assessments.

		Time point				
		Enrollment	Allocation	Postallocation		
		–*t*_1_	0	Baseline	8 Weeks	16-Week follow-up
**Enrollment**						
	Eligibility screen	✓				
	Informed consent	✓				
	Allocation					
**Interventions:**						
	Behavioral intervention				✓	
**Assessments:**						
	Lung function			✓		
	PADL^a^ and sedentary behavior	✓			✓	✓
	Asthma Control	✓			✓	✓
	Asthma exacerbations			✓	✓	✓
	Quality of life			✓	✓	✓
	Anxiety and depression			✓	✓	✓
	Barriers to perform PADL			✓	✓	✓
	Behavioral stage for PADL			✓	✓	✓
	Anthropometric data			✓	✓	✓
	Body composition			✓	✓	✓

^a^PADL: physical activity in daily life.

### Outcome Measures

The primary outcome will be PADL (the number of steps per day) using an activity monitor (by actigraphy). Secondary outcomes will include clinical asthma control, quality of life, body composition and anthropometric data, anxiety and depression levels, asthma exacerbations, physical activity barriers, and behavioral stages for physical activity. This will be a single-blinded (outcome assessor) study in which assessments will be performed before, immediately after, and 16 weeks later.

#### Primary Outcome: PADL

PADL will be objectively assessed using an activity monitor (ActiGraph GT9X), a device that monitors frequency, intensity, and duration [27] in real time and is considered a sensitive and reliable method [28,29]. This triaxial monitor provides measurements of the amount and intensity of PADL and the monitoring of posture [27,30] through variations in acceleration. The “counts” obtained in a given period of time are linearly related to the intensity of the patient’s physical activity in this period [29]. Individuals will be instructed to use the accelerometer, attached to the hip using an elastic belt, for 7 consecutive days. All accelerometers will be initialized to collect data in 60 “epochs” on the 3 axes using ActiLife (version 6.9.5; ActiGraph) software. The following data will be later analyzed using the same software: the number of daily steps and the time spent in sedentary, light, moderate, and vigorous states of activity. Moderate to vigorous physical activity will also be evaluated. Sedentary behavior will be assessed using the same activity monitor.

#### Secondary Outcomes

##### Asthma Clinical Control

Clinical asthma control will be assessed using the ACQ, a reliable and validated tool [16,31] that comprises 7 questions [16]. Five questions are related to asthma symptoms (waking up at night, activity limitation, shortness of breath, and wheezing), 1 question is related to rescue medication (short-acting β_2_-agonist use), and 1 question is related to lung function (forced expiratory volume prebronchodilator, in percentage of predicted). The questions are scored on a 7-point scale ranging from 0 (no limitation) to 6 (maximum limitation), and the total score is the average of the 7 items, ranging from 0 (totally controlled) to 6 (severely uncontrolled). A cut-off value ≥1.5 indicates poorly controlled asthma, 0.75-1.5 indicates partially controlled asthma, and ≤0.75 indicates fully controlled asthma [16]. A change of at least 0.5 points in the ACQ score is considered clinically significant [32].

##### Asthma-Related Quality of Life

Health-related quality of life will be evaluated using the Asthma Quality of Life Questionnaire (AQLQ) [33,34]. This questionnaire consists of 4 domains that assess the last 2 weeks: activity limitation, symptoms, emotional function, and environmental stimulation. The AQLQ score ranges from 0 to 7; the higher the score is, the better the quality of life. A difference of 0.5 points is considered clinically significant [35].

##### Body Composition and Anthropometric Indexes

Octopolar InBody 720 equipment (Biospace) will be used. The weight, fat mass, fat-free mass, visceral adiposity area, and skeletal muscle mass will be calculated. The InBody 720 uses 8 electrodes to assess body composition according to total body water, proteins, minerals, and fat mass. The contact points to connect the electrodes will be cleaned with an electrolytic cloth, according to the manufacturer’s instructions. Data will be electronically imported into Microsoft Excel using Lookin’Body 3.0 software (Biospace). Anthropometric data, including height, body weight (Filizola), abdominal, waist, and hip circumference, and waist-to-hip ratio will be measured according to standardized protocols [36,37]. BMI will be calculated by dividing body weight (in kg) by height (in m^2^) [38].

##### Anxiety and Depression

The Hospital Anxiety and Depression Scale (HADS) will be used to assess anxiety and depression symptoms [39]. The HADS is composed of 14 questions divided into 2 subscales, anxiety and depression (7 questions each). Each question ranges from 0 to 3, with a maximum score of 21 points for each subscale. In this study, a cut-off score (≥8) will be used to classify the presence or absence of symptoms of anxiety or depression [40].

##### Asthma Exacerbations

Asthma exacerbation is defined as an event in which a patient requires urgent action and needs to change the pharmacological treatment [41]. During this study, at least one of the following criteria will be used to define an exacerbation: the use of ≥4 puffs of rescue medication per 24 hours during a 48-hour period, a need for the administration of systemic corticosteroids, an unscheduled medical appointment, and either a visit to the emergency room or hospitalization [21,25,41].

##### The Behavioral Stage for Physical Activity

The assessment of “readiness to change” will be graded using a questionnaire to evaluate the following behavioral stages for physical activity: *Precontemplation*, *Contemplation*, *Preparation*, *Action,* and *Maintenance* [24,42]. The data will be presented in frequencies.

##### Barriers to Performing Physical Activity

Barriers to PADL practice will be assessed using a questionnaire that contains the most common barriers in adults [43,44]. The options to answer each question included: never, rarely, sometimes, almost always, and always, and each item score varies from 0 to 4. Higher scores indicate higher barriers.

### Sample Size Calculation

A minimum number of 17 individuals in at least one of the groups (responder or nonresponder) was established to detect a difference of 2500 (SD 2500) steps [45] in the primary outcome (α=5%, 1 – β=80%). A sample of 100 participants was established, considering the percentage of nonresponders (17%) as previously observed [5]. Clinical improvement in the ACQ score (≥0.5) [3,32] will be considered a secondary outcome.

### Statistical Analysis Planning

Individuals will be stratified as “responders” and “nonresponders” based on an increase of ≥2500 steps [45]. The Kolmogorov-Smirnov test will be used to assess data normality. Cluster analysis will be used to characterize the groups according to individual characteristics. Cluster analysis will be carried out in 2 stages: hierarchical analysis using the Ward method to determine the number of clusters and k-means analysis to group the clusters [46]. Comparisons of clinical, anthropometric, and psychosocial data obtained in the responder and nonresponder groups will be conducted using the 2-tailed *t* test or Mann-Whitney *U* test for continuous variables and the chi-square test for categorical variables. To determine the independent factors associated with the level of PADL (dependent variable, considering clinical control, smoking, obesity, anxiety, and depression as independent variables), multiple linear regression analysis (stepwise forward) will be used. The intra- and between-group comparison at baseline, short term, and medium term will be assessed using the 2-way ANOVA with repeated measures test. The significance level will be adjusted to 5% (*P*<.05) for all tests. If necessary, intention-to-treat analysis will be performed, as described earlier. SPSS Statistics (version 22.0; IBM Corp) will be used for statistical analysis. Data integrity will be monitored by regularly scrutinizing data files for omissions and errors. Participants will be given an anonymous study ID to protect confidentiality, and only study investigators will have access to the final trial data set.

## Results

In December 2021, the clinical trial registration was approved. Recruitment and data collection for the trial is ongoing, and the results of this study are likely to be published in late 2024.

## Discussion

### Potential Impact and Significance of the Study

The role of exercise training in individuals with moderate to severe asthma has been widely studied in recent years [47,48]. Aerobic exercise training improves physical fitness, health-related quality of life, asthma control, and lung function in this population [46,49]. However, despite the beneficial effects of exercise training, little is known about the behavioral modifications needed to achieve a more active lifestyle [4], and studies have been conducted to address behavioral interventions. Very recently, it was demonstrated that a behavioral intervention aimed at increasing PADL modified the behavior of patients with asthma, resulting in an increase in PADL and improvement in symptoms of the disease [5]. This protocol was developed to identify the characteristics of individuals who respond and do not respond to this type of intervention, which may help guide clinical practice.

### Strengths and Limitations of the Study

In addition to the potential to identify the characteristics of responders and nonresponders, this study will also follow up 4 months after training, considered the medium term. These results will allow us to determine if behavioral changes are maintained in the responders over time. The assessment of PADL using accelerometers as a primary outcome is a study strength because it is an objective outcome measure, thus decreasing the risk of bias. The fact that the study will be performed in only 1 hospital center can be considered a limitation.

### Contribution and Clinical Applicability

To our knowledge, this will be the first study to assess the characteristics of responders and nonresponders to a behavioral intervention aimed at increasing PADL in adults with asthma. Therefore, the results obtained in the proposed protocol may provide vital information for health care professionals to refer patients with asthma to more specific intervention approaches (behavioral or otherwise) based on their profile as potential responders or nonresponders. Furthermore, this study may clarify whether the potential gains obtained in the short term after the intervention are maintained in the medium term in individuals with moderate to severe asthma.

### Conclusion

The behavioral intervention to increase PADL may promote different effects according to the clinical characteristics of the individuals, including asthma control, age, anxiety and depression levels, obesity, and several comorbidities. Furthermore, identifying individuals who respond or do not respond to behavioral interventions to increase PADL will help clinicians prescribe specific interventions to adults with asthma.

## Abbreviations

ACQ: Asthma Control Questionnaire
AQLQ: Asthma Quality of Life Questionnaire
GINA: Global Initiative for Asthma
HADS: Hospital Anxiety and Depression Scale
PADL: physical activity in daily life
SPIRIT: Standard Protocol Items: Recommendations for Interventional Trials
WHO: World Health Organization

## Data Availability

The data obtained in this study will be available from the corresponding author (CRFC) upon reasonable request.
